# Molecular ruler of the attachment organelle in *Mycoplasma pneumoniae*

**DOI:** 10.1371/journal.ppat.1009621

**Published:** 2021-06-10

**Authors:** Daisuke Nakane, Kohki Murata, Tsuyoshi Kenri, Keigo Shibayama, Takayuki Nishizaka

**Affiliations:** 1 Department of Engineering Science, Graduate School of Informatics and Engineering, The University of Electro-Communications, Tokyo, Japan; 2 Department of Physics, Gakushuin University, Tokyo, Japan; 3 Department of Bacteriology II, National Institute of Infectious Diseases, Tokyo, Japan; University of Massachusetts Medical School, UNITED STATES

## Abstract

Length control is a fundamental requirement for molecular architecture. Even small wall-less bacteria have specially developed macro-molecular structures to support their survival. *Mycoplasma pneumoniae*, a human pathogen, forms a polar extension called an attachment organelle, which mediates cell division, cytadherence, and cell movement at host cell surface. This characteristic ultrastructure has a constant size of 250–300 nm, but its design principle remains unclear. In this study, we constructed several mutants by genetic manipulation to increase or decrease coiled-coil regions of HMW2, a major component protein of 200 kDa aligned in parallel along the cell axis. HMW2-engineered mutants produced both long and short attachment organelles, which we quantified by transmission electron microscopy and fluorescent microscopy with nano-meter precision. This simple design of HMW2 acting as a molecular ruler for the attachment organelle should provide an insight into bacterial cellular organization and its function for their parasitic lifestyles.

## Author summary

*Mycoplasma pneumoniae*, a pathogen of “walking pneumonia”, have a membrane protrusion with a precise length of 250–300 nm specially developed to support their parasitic lifestyles. To date, however, there has been no report focusing on the potential length-control mechanisms of this characteristic architecture called an attachment organelle. Here, we found that the coiled-coil domains of the 200-kDa protein HMW2 are aligned in parallel along the cell axis, and acts as a molecular ruler by the assembly into a physical scaffold. The molecular ruler could be engineered by genetic modification to produce both longer and shorter attachment organelle. The analyses of the length-controlled mutant highlight a simple design principle of cellular organization in a small bacterium.

## Introduction

**Fig 1:**
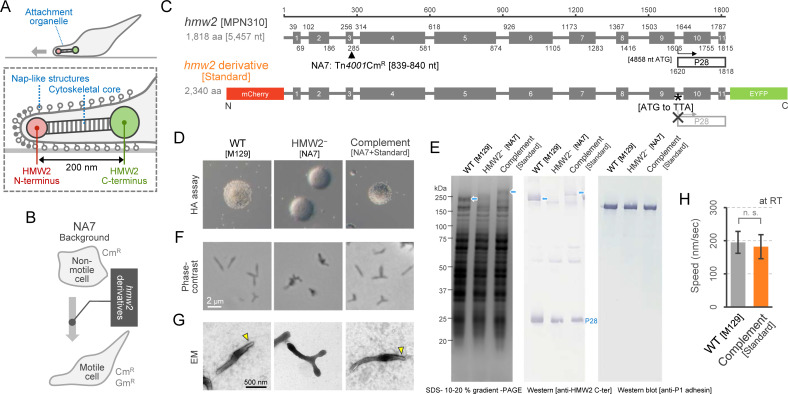
Characterization of an *hmw2*-deficient mutant (NA7) and its complementation. (A) Schematics of *M*. *pnenumoniae* attachment organelle of. (B) Schematic illustration of the NA7 strain complemented with the *hmw2*-derivative gene. (C) Construction of an *hmw2*-derivative gene. Top: original *hmw2*. Bottom: *hmw2*-derivative gene. The gray boxes show 11 domains of the coiled-coil predicted by COILS [41]. The transposon insertion site in the NA7 mutant was indicated by the black triangle. P28 protein derived from internal translation of the *hmw2* [18,21] was presented around the 3´ end. The asterisk shows the codon substitution to disturb the internal translation of P28. (D) Hemadsorption assay using sheep blood cells. (E) Protein profiles. *Left*: Whole cell lysate was subjected to SDS-PAGE, and stained by CBB. *Middle and Right*: Western blot using antiserum against HMW2 [20] and anti-P1 monoclonal antibody [33] used at the dilutions of 1:1,000 and 1:10,000, respectively. The bands of HMW2 and its derivative was indicated by blue arrows. (F) Cell images under phase-contrast microscopy. (G) Cell images under negative-staining electron microscopy. The attachment organelle was indicated by yellow arrowheads. (H) Speed of gliding motility under phase-contrast microscopy at RT. The average and SD were shown (n = 30). Statistical analysis followed by *t*-test are shown; n. s. no significance.

*Mycoplasma* is a small parasitic bacterium without cell wall, and is used as a platform for synthetic biology due to its small genome size [1]. Despite their minimalistic and simple life form, these organisms have developed various macro-molecular structures to support their parasitic lifestyle [2]. In the case of *Mycoplasma pneumoniae*, a common pathogen of community acquired pneumonia, the cell forms a polar extension called attachment organelle, which is required for a wide variety of cellular processes, including cell division, cytadherence at the host cell surfaces [2,3], and gliding motility, i.e., a surface-associated cell movement [4,5]. This characteristic ultrastructure is covered with surface nap-like structures and supported by an internal rod-like cytoskeletal core, which has a consistent size of with a precise length of 250–300 nm along the cell axis (Fig 1A) [6–9]. However, the principles underlying macromolecular assembly remain unclear, one possible explanation for this precise length control is that a molecular ruler exists.

Several length-control mechanisms of biological architecture, such as those of bacteriophage tails, the hook of the bacterial flagellum, the needle of the injectisome of many Gram-negative bacteria, and eukaryotic cilia, have evolved independently [10–16]. However, no studies have focused on the potential length-control mechanisms of attachment organelles in *Mycoplasma*. Previous studies, including genome analyses, have identified dozens of genes as protein components for the attachment organelle, which are only found in related species in *Mycoplasma* [9,17]. One of the major structural components is the high molecular weight protein HMW2, which is predicted to have 11 coiled-coil domains that span 1,257 of the total 1,818 amino acid residues [18]. Recently, we found that HMW2 is localized from the N-terminus at the distal part to the C-terminus at the proximal part of the attachment organelle with a length of 200 nm [9] (Fig 1A). Considering that the predicted α-helix regions of HMW2 are aligned in parallel along the longest cell axis [19], the total length was calculated to be 190 nm as a single chain. This may lead to a simple model in which the size of the coiled-coil domains in HMW2 control the length of the attachment organelle as a structural scaffold. To test this hypothesis, we constructed several mutants by the engineering of HMW2 with insertion or deletion of putative coiled-coil domains, and investigated the length and the function of this molecular architecture.

## Results

### Construction of HMW2 modified strains

We constructed HMW2-derivative expressing *M*. *pneumoniae* strains by the following procedures (Fig 1B, see also the Materials and Methods section for more detail). First, we created an *hmw2*-deficient mutant by a transposon mutagenesis (Fig 1C, *top*), i.e., a mutant designated as NA7 that did not exhibit hemadsorption activity (Fig 1D). An insertion of transposon Tn*4001*Cm was detected between nucleotides 839 and 840 of *hmw2*. We analyzed NA7 cells by SDS-PAGE and Western blot using anti-HMW2 serum and confirmed that full length HMW2 was not detected in this mutant (Fig 1E), although P28 protein, a product from the internal start codon at nucleotide 4858 of *hmw2*, was still present [20]. We also observed the cell morphology of NA7 under optical and electron microscopy, and confirmed the loss of polar extension from the cell body, suggesting a failure of attachment organelle formation (Fig 1F and 1G). These phenotypes of the *hmw2*-deficient mutant were consistent with previous reports [21,22]. Then, we constructed an *hmw2*-derivative gene to complement and restore the phenotype of NA7. This *hmw2*-derivative gene had the full-length sequence of the original *hmw2*, fused with *mcherry* and *eyfp* fluorescent protein genes at the 5´ and 3´ end, respectively (Fig 1C). We also introduced a substitution from ATG to TTA at the 1,620^th^ codon of the original *hmw2* to disturb internal translation of P28 protein (Fig 1C). We introduced this *hmw2*-derivative into NA7 by using a Tn*4001*Gm vector (Fig 1B). Analysis of the NA7 strain complemented with *hmw2*-derivative showed expression of HMW2-derivative protein (Fig 1E), and restoration of the *hmw2*-deficient phenotypes including the attachment organelle formation, cytadherence, and gliding motility to levels similar to those of the wildtype (WT), i.e., *M*. *pneumoniae* M129 strain (Fig 1D, 1F, 1G, and 1H). Although the HMW2-derivative protein carried mCherry and EYFP tags at the N and C termini, there were no significant phenotype differences between the complemented NA7 strain and WT. Therefore, this complemented strain was used as a ‘standard’ in the following experiment (Fig 1C, *bottom*).

**Fig 2:**
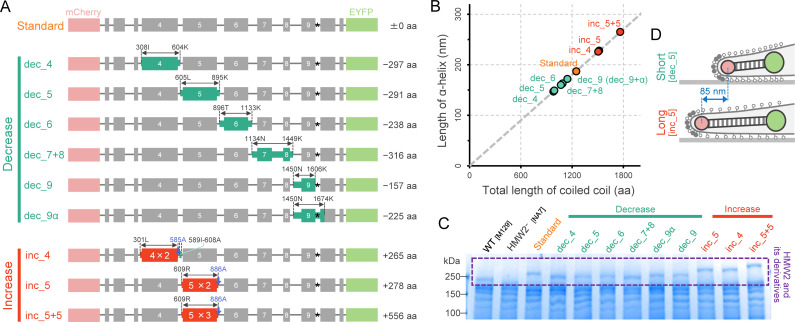
Engineering of HMW2. (A) Construction of the size-modified HMW2-derivatives. The decreased and increased parts are were in green and red, respectively. The difference of amino acids number was presented at the right. All derivatives were fused with mCherry and EYFP at the N- and C-termini, respectively, and have the codon substitution to disturb the internal translation of P28. (B) Length estimation of the size-modified HMW2-derivatives. The length of α-helix was calculated by the total amino acid numbers of predicted coiled-coil domains in each HMW2 derivative. (C) Protein profiles of WT and HMW2-derivative mutants. The entire cell lysate was subjected to SDS-PAGE, and stained by CBB. The molecular mass was shown on the left. The protein bands from *hmw2* derivatives were shown in a dashed box (see also S1 Fig). (D) Schematics of the length-controlled attachment organelle in the size-modified HMW2-derivative mutants.

**Fig 3:**
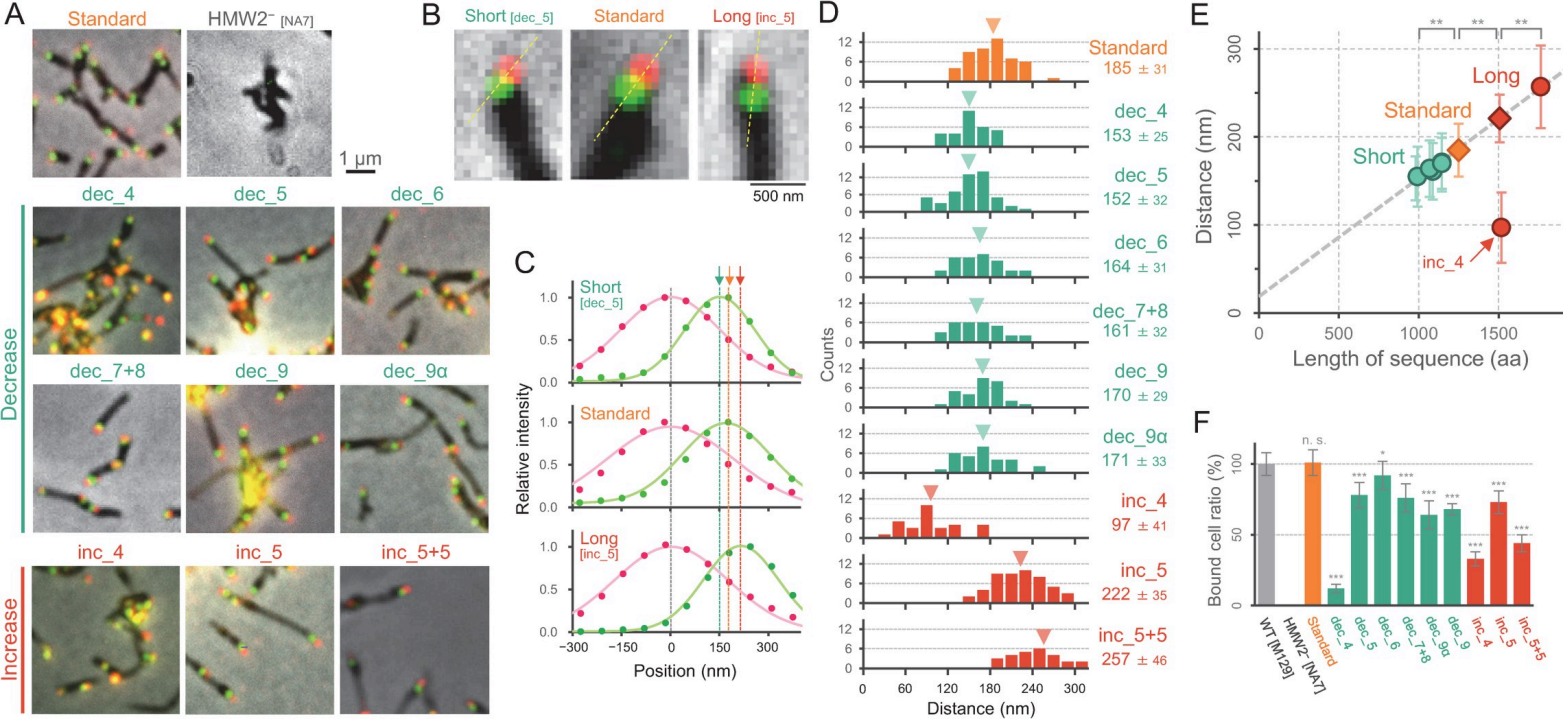
Localization of the size-modified HMW2 derivatives in the attachment organelle. (A) Cell images. Fluorescence and phase-contrast images were merged. (B) Magnified images of the attachment organelle in dec_5, standard, and inc_5. (C) Measurement of peak positions between mCherry and EYFP signals. Fluorescent intensities along the dashed line in B were plotted with Gaussian distribution. (D) Distribution of the distance between two signals. The average and SD of 30 cells were presented, and the averrage was marked by a triangle in each panel. (E) Relationship of the distance between mCherry and EYFP signals, and the total amino acid numbers of coiled-coil domains in HMW2. The average and SD in panel D were plotted. Except for the data from inc_4 strain, the linear fitting was shown as a dashed line (see in S3 Fig for more detail). (F) The ratio of cells bound to glass. The number of cells in 62 × 35 μm^2^ area was counted after washing unbound cells at RT. The average and SD of 12 independent experiments were presented. Statistical analysis followed by *t*-test are shown; **p 0.01, n. s. no significance.

We constructed nine size-modified *hmw2*-derivative genes as listed in Fig 2A and S1 and S2 Tables. These short and long *hmw2*-derivatives were designed by deletion and insertion of the predicted coiled-coil domain. Six short derivatives, designated as dec_4, dec_5, dec_6, dec_7+8, dec_9, and dec_9α, had deletions of 4^th^, 5^th^, 6^th^, 7-8^th^, 9^th^, and 9^th^+α domains of the predicted coiled-coil, respectively. The +α indicated an additional deletion of 68 aa at the linker part between the coiled-coil domains. Three long derivatives, designated as inc_4, inc_5, and inc_5+5 had double 4^th^, double 5^th^, and triple 5^th^ domains, respectively. Total amino acid numbers of the predicted coiled-coil domains of these nine derivatives ranged from 981 to 1,763, and the axial length of the α-helix calculated from amino acid numbers ranged from 147 to 265 nm (Fig 2B and S3 Table). These *hmw2*-derivative genes were introduced to NA7, and we analyzed the nine complemented strains by SDS-PAGE, confirming that expression levels of nine HMW2 derivatives were comparable to that of the native HMW2 in WT. As expected, the protein bands of HMW2 derivatives were shifted to the higher molecular weight in SDS-PAGE, because the molecular size was increased by the fusion of mCherry and EYFP (Figs 2C and S1). We also analyzed the nine strains by Western blot against P1 (S1 Fig), which is a major component of the surface nap-like structure of the attachment organelle [23,24], and confirmed that P1 expression level showed no significant change compared to that of WT. Additionally, we also observed the cell morphology of these strains under optical microscopy, and confirmed that they restored the attachment organelle formation and cytadherence activity (Figs 3A and S2). Thus, the HMW2 derivatives were largely functional as attachment organelle component and compensate the native HMW2. Focusing on dec_5 and inc_5 as putative short and long HMW2-derivative mutants, the difference in axial length of α-helix was estimated to be 85 nm (Fig 2D).

### Length control of the attachment organelle

To substantiate the hypothesis that the length of the attachment organelle is controlled by the size of HMW2, we observed fluorescent signals from the HMW2 derivatives in living cells. As the cell attached to the glass surface did not show clear gliding motility during a short observation period at room temperature (RT) of 25°C [9], we took the fluorescent images without chemical fixation. A clear signal of mCherry and EYFP was detected at the distal and proximal part at the attachment organelle, respectively (Fig 3A), suggesting that the mutant keeps the polarity to assemble both N- and C- termini of HMW2 in the same orientation. We picked the individual cell bound on a glass surface for further analysis of the fluorescent localization. We did not include the data from other cells which was branched at the cell pole or detected multiple signals in single cell. The fluorescence peak position was precisely determined by fitting with the Gaussian distribution (Fig 3B and 3C), with the distance between two peaks being 185 ± 31 nm in the standard strain as previously observed [9], but decreasing to 152 ± 32 nm in the dec_5 strain and increasing to 222 ± 35 nm in the inc_5 strain. We measured the distance between fluorescent peaks in all mutants (Fig 3D), and plotted them as a function for the total amino acid numbers of the predicted coiled-coil domains in each HMW2 derivative (Figs 3E and S3). As expected, the graph showed a proportional relationship with a slope of linear approximation of 0.13 nm per amino acid, suggesting that the attachment organelle length was determined by the number of amino acids in the α-helix region of HMW2. A the only one exception, inc_4 was a long HMW2 derivative with the additional 4^th^ coiled-coil domain, but the distance between peaks was 97 ± 41 nm, about a half that of the standard strain (Fig 3E). We could not have a clear explanation for this short distance. One possibility is that the long HMW2 in inc_4 is incorporated into the attachment organelle with an alternative conformational state.

**Fig 4:**
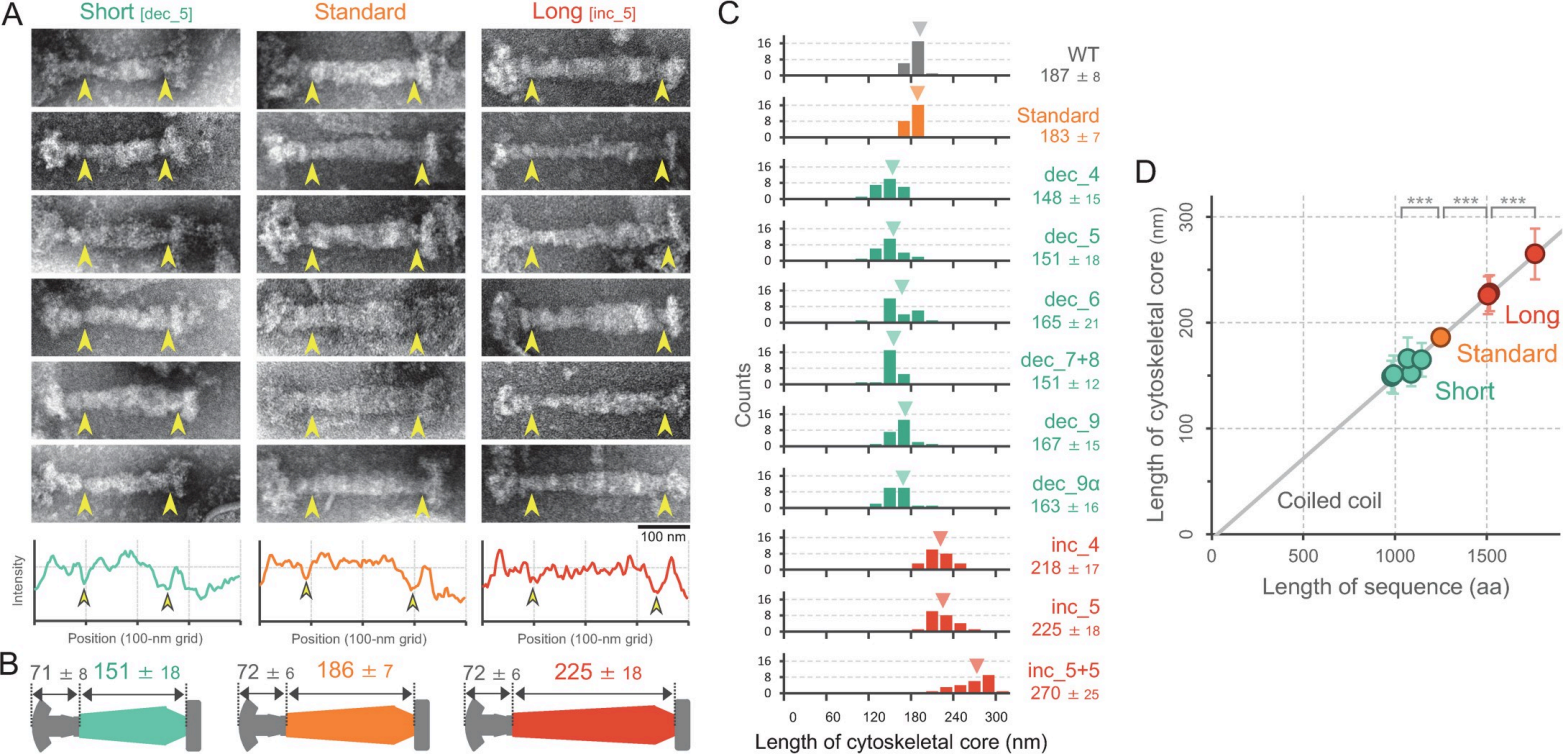
Cytoskeletal core of the size-modified HMW2-derivative mutants. (A) *Top*: EM images. Cells were treated with 1% Triton X-100, and the remaining structure was observed under negative-staining EM. The distance between the structural boundary indicated by the yellow arrowheads was measured as the length of the cytoskeletal core. *Bottom*: The intensity profiles of cytoskeletal structure along the longer axis were measured, and the average from 6 image were shown. The structural boundaries at the distal and the proximal parts were marked by yellow arrowheads. (B) Schematics of the isolated cytoskeletal cores. (C) Length distribution of the cytoskeletal core. The average and SD of 24 structures were presented, and the average was marked by a triangle in each panel. (D) Relationship between the cytoskeletal core length and the total amino acid numbers of coiled-coil domains in HMW2 derivatives. The average and SD in panel C were plotted. The linear fit was shown as a gray line. All data were analyzed from images of the *bold*-type structure. Statistical analysis followed by *t*-test are shown; ***p 0.001.

To directly visualize the rod-like cytoskeletal core, we isolated the structure through a treatment with detergent Triton X-100 [9], and observed it under an electron microscope (Fig 4). As previously suggested, the electron microscopy (EM) images of the cytoskeletal core structure could be divided into three parts: a terminal button at the distal end, central paired plates, and a bowl (wheel) complex at the proximal end [9,25,26]. In the size-modified HMW2-derivative strains, structure of the terminal button and the bowl complex did not change, but the paired plate showed clear differences in an axial length compared to those of the original strain (Fig 4A). We measured the length between the structural boundary from the distal to the proximal part of constricting regions (represented by the yellow arrowheads in Fig 4A), and found that the average length was 186 ± 8 nm in the standard strain, but decreased to 145 ± 18 nm in the dec_5 strain and increased to 227 ± 15 nm in the inc_5 strain (Fig 4B). In all nine HMW2 derivatives, the cytoskeletal core (Fig 4C) showed a proportional relationship with the total amino acid numbers of the predicted coiled-coil domains (Figs 4D and S3), with a slope of 0.15 nm per amino acid, which was in good agreement with the translation along the longitudinal axis of the α-helix. These results clearly showed that the domains from the 4^th^ to 9^th^ coiled-coil in HMW2 were responsible for length control of the cytoskeletal core structure as a structural scaffold by α-helix formation. Meanwhile, the cytoskeletal core showed structural variation categorized as *bold* and *slim* types, that have set down on the surface on their broad side and on their narrow side, respectively [9], and we observed this length-control feature in both types (Figs 4D and S4). In addition, we directly measured the length of a polar extension of cell which have electron-dense region, presumably as a length of the attachment organelle (S5 Fig). The average length was decreased to be 240 ± 15 nm in the dec_5 strain, and increased to 331 ± 15 nm in inc_5 strain, while the standard strain length was comparable to that of WT as 284 ± 11 nm. This length was consistent with the length of the attachment organelle previously observed by cryoEM [7,27,28]. We also observed the localization of P1 protein, a major component of nap, by immuno-gold EM. The distribution of the gold particles showed short and long tendency at the attachment organelle in dec_5 and inc_5, respectively. Accordingly, the most straightforward interpretation is that HMW2 functions as a molecular ruler to determine the length of the attachment organelle by the assembly of cytoskeletal core.

### Relationship between gliding motility and length of the attachment organelle

How does the engineering of HMW2 protein affect its function? These mutants did not increase or decrease the total amount of surface protein P1 in the attachment organelle (S1 and S6 Figs), and did not show obvious defects in binding activity to a glass surface (Fig 3F). The attachment organelle in the HMW2-derivative mutants appeared to be capable of generating the propulsion force required for gliding motility.

To precisely measure the cell movement, we observed the single cell behavior under an optical microscope by the following three procedures (see more detail in Materials and Methods). First, we passed the cell culture through a needle and filtered to generate single cell suspension [29,30]. Although cells often form cell aggregates in the growth culture, the microscopic image of this suspension showed many individual cells that adhered to the glass surface. Second, we used a serum without heat inactivation. Although the heat inactivation of horse serum at 56°C is essential to destroy complement activity for the growth medium, we found that gliding motility improved on a glass surface pre-treated with the non-heated serum. We assumed that the heat inactivation also destroys heat labile factors in the serum, which are involved in the motility, such as sialoprotein as a binding target [31,32]. In this study, the non-heated serum was used only for the observation of single cell movement under the optical microscopy. Third, we observed the cell at RT rather than their optimal growth temperature[33]. During cell incubation at 37°C, the sequential images of cells showed thermal drifts because of the heating of a microscope stage. As this is a problem for the precise measurement of cell behavior, we recorded cell gliding motility without stage heating. With the non-heated serum, the cell showed clear gliding motility even at RT (S1 Movie). The gliding speed in WT was 195 ± 33 nm s^-1^ at RT (Fig 1H), which was 70% slower than that at 37°C [34].

**Fig 5:**
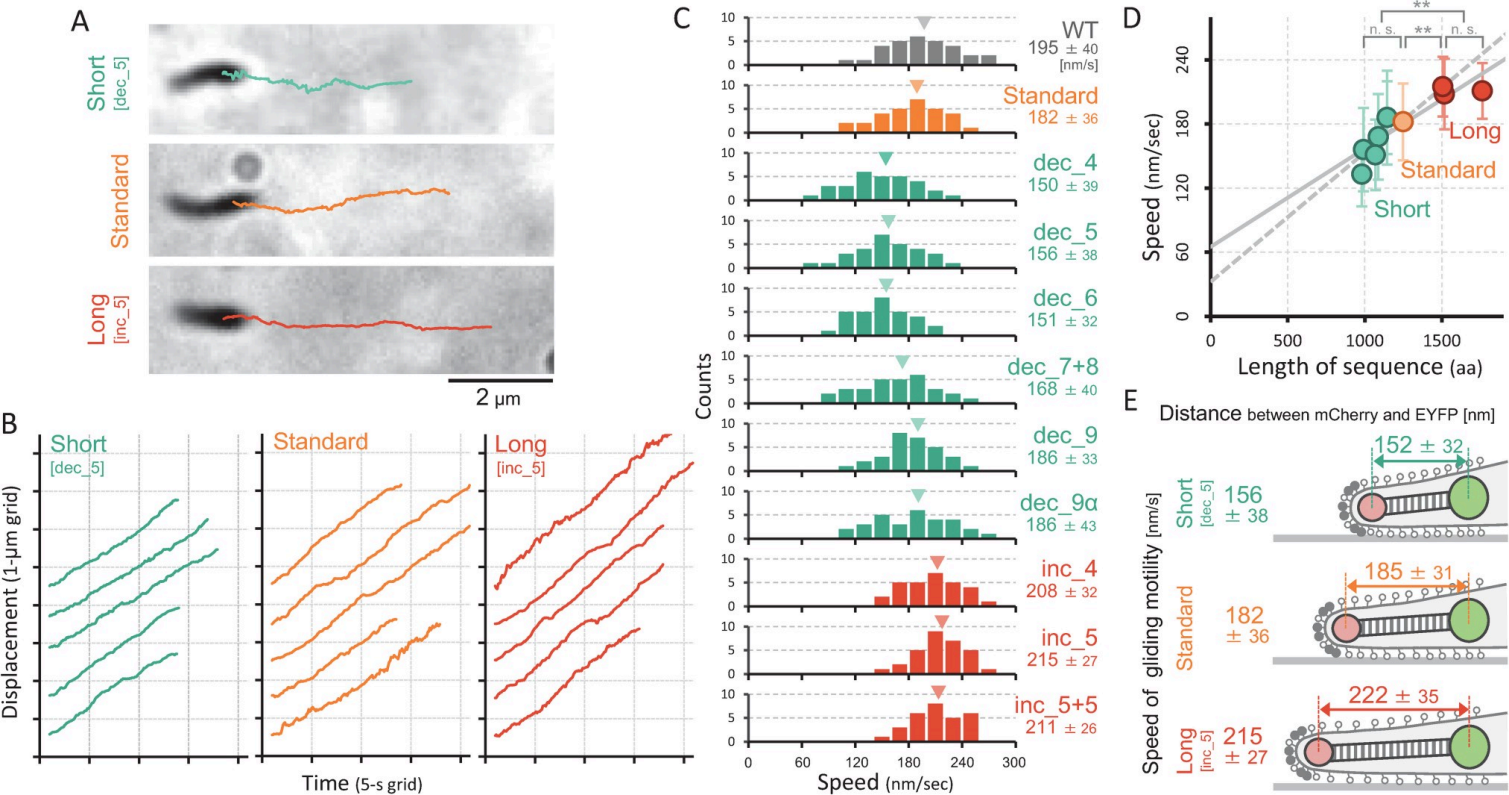
Gliding motility of the size-modified HMW2-derivative mutants. (A) Cell images and trajectories for 8.5 s. (B) Time course of the displacement of straightly moving cells. (C) Distribution of the gliding speed. The average and SD of 30 cells were presented in each panel. (D) Relationship between the gliding speed and the total amino acid sequence of coiled-coil domains in HMW2 derivatives. The average and SD in panel C were plotted. The solid and dashed lines showed the linear fit of all strains and the data excluding inc_5+5, respectively. Statistical analysis followed by *t*-test are shown; **p 0.01, n. s. no significance. (E) Schematic illustration of the length-controlled attachment organelle and gliding speed.

Next, we examined gliding motility in the size-modified HMW2-derivative strains under an optical microscopy. We selected the straightly moving cells with less than 0.19 of curvature in a trajectory for precise measurements (Fig 5A). Cell movement was projected to the axis of cell displacement duration (Fig 5B), and the linear fitting of the slope was considered the speed of gliding motility. Importantly, the average speed was decreased to be 156 ± 38 nm s^-1^ in the dec_5 strain, and increased to 215 ± 27 nm s^-1^ in the inc_5 strain, while the standard strain speed was comparable to that of WT (Fig 5C and S2 Movie). We measured the average gliding speed in all nine HMW2 derivative strains (Fig 5C), and plotted them as a function of the total amino acid numbers of the predicted coiled-coil domains in each HMW2 derivative (Figs 5D and S3), showing a positive correlation. Additionally, the speed rate increment was lowest in the largest HMW2 derivative strain designated as inc_5+5, which had the triple 5^th^ coiled-coil domains with additional 560 amino acids in total. This positive correlation may have the upper speed limit at around 220 nm s^-1^ at RT.

Previously, we showed that the gliding motility in *Mycoplasma mobile* shows stepwise displacement of 70 nm, presumably by the repeated cycle of conformational change of the surface “leg” structure [35], where the molecular system of *M*. *mobile* for the gliding motility is phylogenetically distant from that of *M*. *pneumoniae* [5]. To test the hypothesis that *M*. *pneumoniae* cell shows stepping motion, we performed the simultaneous observation of two fluorescent proteins at the attachment organelle during the cell movement with high precision colocalization microscopy at a time resolution of 50 Hz (S7 Fig). However, we could not detect such large stepwise displacement in *M*. *pneumoniae* by simultaneous observation of mCherry at the N-terminus and EYFP at the C-terminus in HMW2 (N = 20 cells).

## Discussion

Here, we showed direct evidence that the HMW2 protein coiled-coil domain works as a molecular ruler, which could be modified by genetic engineering to produce both longer and shorter attachment organelles (Figs 2, 3, 4 and S3). Unlike the length-controlling system in the flagellar T3S system [11,15], *M*. *pneumoniae* molecular ruler provides a physical scaffold for assembly of the cytoskeletal core, and had an impact on its gliding motility function. Assuming that the cytoskeletal core undergoes inchworm-like movement to push the tip of the cell forward in steps [6,7,26,28], we expect that a mutant with a longer cytoskeletal core would show faster cell movement. This idea is supported by the positive correlation between HMW2 size and average gliding speed (Fig 5 and S3 Table). It was also expected that the cytoskeletal core produced the repeated contraction and extension cycle where the front end always leads while the back end follows. However, we could not detect stepwise displacement of more than dozens of nanometers (S7 Fig), suggesting that the cytoskeletal core does not directly generate large conformational changes.

Then, why does the cell move faster with a longer cytoskeletal core? Surprisingly, the total amount of P1 protein in the attachment organelle was almost the same in the length-controlled HMW2 mutants (S1 and S6 Figs), while the length of the attachment organelle was different about 90 nm in length between short and long strains (S5 Fig). Considering that the nap-like structures are aligned in the limited surface space of 280 nm in WT [6,7,28], the density is increased to be 1.18 in dec_5 and decreased to be 0.86 in inc_5. This implies the lower density of nap shows faster gliding motility (S6 Fig). In the case of the *in vitro* gliding assay of eukaryotic motor proteins, the gliding velocity is limited by a balance between the gliding force and the frictional drag, which is explained by the basic assumption that the motor can take three states: detached, driving, and holding states [36,37]. As the nap-like structures composed of P1/P90/P40 complex interacts with sialic acid, a binding target at the surface [24,32,38], the same idea might be applicable in *M*. *pneumoniae*. In that case, it is reasonable that the arrangement of the nap-structure per unit length would affect the cooperativity of the propulsion force, which may be a reason for HMW2 acting as a molecular speedometer for gliding motility. Considering that the length of the attachment organelle and the gliding speed varied in related *Mycoplasma* [8], this simple design of HMW2 may have contributed to the adaptation to their parasitic environments and pathogenicity. However, further study is needed to observe the effect of length on the cytadherence and motility of the attachment organelle *in vivo* to test these predictions.

## Materials and methods

### Bacterial growth conditions

*M*. *pneumoniae* M129 (subtype 1) [39] was used as the wild-type strain. Cells were grown in Aluotto or PPLO medium at 37°C in a tissue culture flask [9,40]. *Escherichia coli* strains DB3.1, DH5α, and JM83 were used for plasmid construction. For the selection and maintenance of *M*. *pneumoniae* and *E*. *coli* antibiotic-resistant strains, gentamicin (Gm), chloramphenicol (Cm), ampicillin (Ap), and kanamycin (Km), were used at 18, 15, 50, and 50 μg ml^−1^.

### Strain construction

The oligo DNA primers, plasmids, and bacterial strains used in this study were listed in S1, S2, and S3 Tables. All HMW2-derivative expressing strains were constructed by introducing *hmw2*-derivative genes by Tn*4001*Gm transposon vector via electroporation to NA7 strain (Fig 1B) [42], an HMW2-deficient mutant generated by Tn*4001*Cm insertion into the *hmw2* gene (between nucleotide number 839 and 840) (Fig 1C, *top*). Previously described protocols were used for plasmid construction and transformation of *M*. *pneumoniae* [9]. For mCherry fusion at the N-terminus of HMW2, the *mCherry* gene was amplified from pmCherry plasmid (TaKaRa Bio, Japan) using primers mCh-Not-F and mCh-Not-R, and was inserted at the *Not*I site of pMPN310-E plasmid by using Gibson assembly Master Mix (New England Biolabs, USA), and the resultant plasmid was designated as pKM310-mCh. The internal ATG start codon for P28 protein was converted into TTA by using PrimeSTAR Mutagenesis Basal Kit (TaKaRa Bio) and primers MPN310-full-F and MPN310-full-R. The resultant plasmid was designated as pKM310-standard (Fig 1C, *bottom*). All size-modified HMW2 derivative genes were constructed and expressed based on the pKM310-standard plasmid (Fig 2A). For constructing the short *hmw2* derivatives, part of *hmw2* from pKM310-standard was deleted by using the primers listed in S1 Table and PrimeSTAR Mutagenesis Basal Kit. For constructing the long *hmw2* derivatives, the regions of *hmw2* containing coiled-coil domain(s) were amplified by PCR using the primers listed in S1 Table and inserted into *Xba*I-*Bsi*WI or *Nae*I sites of pKM310-standard plasmid by using a NEBuilder HiFi DNA Assembly Cloning kit (New England Biolabs) according to the manufacturer’s instructions. The structures of these *hmw2*-derivative genes were confirmed by sequencing. All *hmw2*-derivative genes fused with *mCherry* at the 5´ end were subcloned into pTK170-D plasmid by Gateway cloning method [9] to fuse with *eyfp* gene at 3´ end, resulting in the pKM170 series of plasmids listed in S2 Table. These pKM170 plasmids were used for transformation of NA7 (S3 Table). Transformants were selected in PPLO broth containing Cm and Gm.

### Cell preparation of gliding motility

In this study, we slightly modified the optimal conditions for the gliding motility previously published [33]. Cells were inoculated in the medium containing 56 μM Phenol Red (Sigma-Aldrich) in a tissue culture flask for 2−3 days at 37°C, and the cell culture was used for the observation of gliding motility when the cell supernatant reached an OD^558^ of 0.55−0.70. The flask was washed twice with PBS/HS, i.e., 10% horse serum (Gibco) in phosphate-buffered saline (PBS) consisting of 75 mM sodium phosphate (pH 7.4) and 68 mM NaCl, and the remaining cells attached to the bottom of the tissue culture flask were scraped in PBS/HS. The suspension was passed though the syringe needle more than 10 times (21G × 38 mm; Terumo), and filtrated with a syringe-driven filter unit (Millex LH 0.45 μm; Millipore) [29,30]. The cell suspension was poured into a flow chamber assembled by taping coverslips that were pre-coated with PBS/HS. After incubation for 10 min at RT, the chamber was washed with PBS/HS, and used for the gliding motility observation. Note that the horse serum for preparing the flow chamber was used without heat-inactivation [34]. The cells that showed straight trajectories were used for the gliding speed analyses. The ratio of cells bound to the glass surface was measured by counting cells within an area of 62 × 35 μm, and normalized to the ratio of WT as 100%.

### Hemadsorption assays

Hemadsorption assays were performed as previously described [19] with minor modifications. Briefly, cells were diluted and inoculated on PPLO agar plates, and incubated for 7 days at 37°C. Sheep blood (Japan Bio Serum) diluted 1:200 in PBS was added to each plate, and incubated for 15 min at 37°C. Plates were washed two times in PBS. Colonies were observed at under a stereo microscope (SZX12, Olympus). Images were captured with a camera (EOS Kiss X8i, Canon).

### Optical microscopy

All measurements were done under an inverted microscope (IX71; Olympus) equipped with a 100× objective lense (UPLSAPO 100×OPH, 1.4 N.A.; Olympus), a CMOS camera (Zyla 4.2; Andor), and an optical table (HAX-0806; JVI). Projection of the image to the camera was made at 65 nm per pixel. Images of cells were acquired by the imaging software (Solis; Andor) as 16-bit images with the CMOS camera, and converted into a sequential TIF file without any compression.

For the observation of mCherry and EYFP localization at the attachment organelle, fluorescent signals were visualized with a filter set (59026; Chroma) and a dual-view imaging system (DV2; Photometrics, 565dcxr; Chroma, FF03-525/50 and BLP01-568R; Semrock, USA). Prior to the data aquation, we observed more than 3 numbers of beads with a size of 200 nm attached to a glass surface, and detected the peak positions by the Gaussian fitting. The bead positions were used as fiducial markers for spatial calibration. We recorded the two independent fluorescent signals from mCherry and EYFP at the same image sensor of camera, and then integrated into one image with pseudo-coloring. A 2 ml culture was grown in a glass-bottom dish (Iwaki, Japan) and observed directly at RT.

For the observation of mCherry and EYFP during the gliding motility, two laser beams (OBIS488 and OBIS561; Coherent) were assembled by a dichroic mirror (LM01-503; Semrock), and introduced into the microscope with a dichroic mirror (Di03-R488/561; Semrock), dual-view imaging system (DV2; Photometrics, 565dcxr; Chroma, FF03-535/50 and BLP01-568R; Semrock) (S7 Fig). The cell was prepared by the gliding motility treatment described the above, and observed under the optical microscopy at RT.

### Electron microscopy

The sample preparation for negative staining EM followed the same protocol as previously described [9]. Carbon-coated EM grids were glow-discharged by a PIB-10 hydrophilic treatment device (Vacuum Device). The cytoskeletal core of each mutant cell was isolated as previously described [9], and the suspension of the isolated core was placed directly onto the carbon-coated grids for 5 min at RT. After removal of the solution, the grid was washed with PBS and stained by 2% ammonium molybdate (vol/vol). For the attachment organelle observations, the cells were suspended in PPLO medium at 30-fold density of the original culture, put on the carbon-coated grids for 10 min at 37°C. The cells were chemically fixed by 1% glutaraldehyde in PBS for 10 min at RT, and washed three times by PBS. For the immunogold EM, the cells bound to EM grids were treated with an anti-P1 monoclonal antibody [33] in PBS containing 2% bovine serum albumin (BSA) and gold-labelled antibody (goat antibody with 10-nm colloidal gold, Sigma) for 30 min at RT, respectively. After removal of the solution, the grid was washed with PBS and stained by 2% ammonium molybdate (vol/vol). Samples were observed under a transmission electron microscope (JEM-1400, JEOL) at 100 kV. EM images were captured by a charge-coupled device camera, and analysed by ImageJ 1.48v.

## Supporting information

S1 Table DNA primers used for plasmid construction in this study. (DOCX)

S2 Table Plasmids used in this study. (DOCX)

S3 Table Size-modified HMW2-derivative mutants in this study and their characterization. (DOCX)

S1 Fig Protein expression in the size-modified HMW2-derivative mutants. (A) Protein profiles of WT and the HMW2-derivative mutants. The wide image of Fig 2C is presented. The cell lysate was subjected to SDS-PAGE, and stained by CBB, and protein concentration of each sample was adjusted to be 10 μg in each lane. The molecular mass was shown on the left. Protein bands of HMW2 derivatives were shown in a dashed box. The HMW2 protein band was not detected in the NA7 mutant. (B) Western blot. Anti-P1 monoclonal antibody was used at a dilution of 1:10,000.
(EPS)

S2 Fig Hemadsorption assays in the size-modified HMW2-derivative mutants. (EPS)

S3 Fig Length and distance of the attachment organelle and speed of gliding motility in the size-modified HMW2-derivative mutants. The data of all mutants was indicated by the dashed line. (A) Relationship between the distance of two fluorescent signals and the total amino acid numbers in HMW2-derivative mutants. (B) Relationship between the length of cytoskeletal core as presented schematically in Fig 4B and total amino acid numbers in HMW2-derivative mutants. (C) Relationship between the speed of gliding motility and total amino acid numbers in HMW2-derivative mutants. (D) Relationship between the gliding speed and the distance of two fluorescent signals. (E) Relationship between the gliding speed and the length of cytoskeletal core as presented schematically in Fig 4B. Gray and purple dashed lines indicated the linear fitting of the total amino acid numbers of coiled-coil domains and the whole sequence of HMW2 derivatives without mCherry or EYFP, respectively.
(EPS)

S4 Fig Cytoskeletal core categorized as *slim* type in the size-modified HMW2-derivative mutants. (A) EM images. Cell were treated with 1% Triton X-100, and the remaining structure was observed under negative-staining EM. (B) Schematics of the isolated cytoskeletal cores. The distance between the yellow arrows in panel A indicated the structural boundary and was measured as the length of the cytoskeletal core. (C) Length distribution of the cytoskeletal core from nine HMW2 mutants. The average and SD of 24 structures were presented, and the average was marked by a triangle in each panel. (D) Relationship between the lengths of the cytoskeletal core and amino acid sequence. The average and SD in panel C were plotted. Linear fittings of the total length of coiled-coil domains and the whole sequence of HMW2 derivatives were shown as gray and purple lines, respectively. Statistical analysis followed by *t*-test are shown; ***p 0.001.
(EPS)

S5 Fig The attachment organelle formation in the size-modified HMW2-derivative mutants. (A) EM images. The length of a polar extension of cell membrane that showed electron-dense region was measued as presented by dashed yellow lines. (B) Distribution in lengh of the polar extension. The average and SD of 20 structures were presented, and the average was marked by a triangle in each panel. (C) Relationship between the length of a polar extension and the total amino acid numbers of HMW2 derivatives. The average and SD in panel B were plotted. Linear fittings of the total length of coiled-coil domains and the whole sequence of HMW2 derivatives were shown as gray and purple lines, respectively. Statistical analysis followed by *t*-test are shown; ***p 0.001. (D) Immunogold EM. The cells were chemically fixed, and labeled by the antibodies against P1 protein and the colloidal 10-nm gold particles. (E) Distribution of the positions of colloidal gold particles measured from the front end of the cell. The data collected from 10 cell images were integrated.
(EPS)

S6 Fig Localization and the protein amount of P1 in the size-modified HMW2-derivative mutants. (A) Immunofluorescence microscopy against anti-P1 antibody. The fluorescent image was merged with a cell image under phase-contrast microscopy. (B) Intensity profiles along the yellow dashed line in panel A. (C) Average of the relative fluorescent intensity. Sum of the intensity at the attachment organelle was measured, and the average and SD from 12 cells were plotted. (D) Schematics of the spatial arrangement of surface nap-structure and internal cytoskeletal core structure in the attachment organelle. Statistical analysis followed by *t*-test are shown; n. s. no significance.
(EPS)

S7 Fig Simultaneous visualization of the N- and C-terminus parts of the size-modified HMW2-derivative mutants. (A) Experimental setup. A dual-view system for mCherry and EYFP at the same sensor of the camera was constructed with dual excitation from lasers of 488 and 561 nm wavelength (see the Materials and Methods section for further detail). (B) Typical example of the fluorescent intensities from a single cell of standard HMW2 strains. The HMW2 derivatives were fused with mCherry at the N-terminus and with EYFP at the C-terminus. The cell showing straight movement was used for further analysis as shown in panel C. (C) Time course of the displacement of EYFP and mCherry. The intensity profile of each fluorescent signal was fitted with Gaussian distribution, and the peak position was plotted.
(EPS)

S1 Movie Field view of gliding motility. The standard strain was observed at RT. The area was 41.6 × 31.2 μm.
(AVI)

S2 Movie Competition assay of gliding motility. Three mutants of dec_5, standard, and inc_5 were aligned from the top, and single cellular behavior was observed at RT. The area was 6.0 × 4.5 μm.
(AVI)
