# Supplementary material for: Molecular ruler of the attachment organelle in Mycoplasma pneumoniae
Source: PLoS Pathog. 2021 Jun 10;17(6):e1009621. doi: 10.1371/journal.ppat.1009621 (PMC8191905; doi:10.1371/journal.ppat.1009621)
Supplement: S1 Table — (DOCX) [file ppat.1009621.s001.docx]

| **S1 Table**  DNA primers used for plasmid construction in this study | | |
| --- | --- | --- |
| Plasmids | Oligonucleotide | Sequence |
| pKM310-mCh | mCh-Not-F | **AGCAGGCTCCGC**GGCCGCCATGGTGAGCAAGGGCGAGGA |
|  | mCh-Not-R | **TATCATTCATGGC**GGCCGCCTTGTACAGCTCGTCCATGC |
| pKM310-standard | MPN310-full-F | **AATGATTTAAACTAT**CCTTACCCATAT |
|  | MPN310-full-R | **ATAGTTTAAATCATT**ACCGTTAATTTT |
| pKM310-dec_4 | MPN310-d4-F | **CTAGCAAGCTGTTGG**AAGCACGTACGC |
|  | MPN310-d4-R | **CCAACAGCTTGCTAG**TTAAATCATCTA |
| pKM310-dec_5 | MPN310-d5-F | **AGCACAAGACAACGC**AAATGCAGCGTTTA |
|  | MPN310-d5-R | **GCGTTGTCTTGTGCT**TTTCTTGTTGTA |
| pKM310-dec_6 | MPN310-d6-F | **ACTTTAAAAACTTTC**AAAAGGCGAGTGA |
|  | MPN310-d6-R | **GAAAGTTTTTAAAGT**GCACTTGGCGC |
| pKM310-dec_78 | MPN310-d78-F | **AGAAGAAGAATCGCT**TGTTTGAATAC |
|  | MPN310-d78-R | **AGCGATTCTTCTTCT**CTAGTAAAAA |
| pKM310-dec_9 | MPN310-d9-F | **CAACAAACACTTATT**TCCACCGTTATTT |
|  | MPN310-d9-R | **AATAAGTGTTTGTTG**TTATCAAACTCACT |
| pKM310-dec_9α | MPN310-d9a-F | **CAACAAACTAGACCA**AATTCAACTCGA |
|  | MPN310-d9a-R | **TGGTCTAGTTTGTTG**TTATCAAACTCACT |
| pKM310-inc_4 | MPN310-dup4-F | **TGATCTAGAA**TTAGATGATTTAACTAGCA |
|  | MPN310-dup4-R | **CTGCGTACG**TGCGTGGTGGTGTTTGTTTTGTT |
| pKM310-inc_5 | MPN310-dup5-F | **GCCGAACATGTAGCC**CGTACGCAGTTTGACAACCG |
|  | MPN310-dup5-R | **TTGGCGCTTAAAGCC**GGCTA |
| pKM310-inc_5+5 | MPN310-dup5-F | **GCCGAACATGTAGCC**CGTACGCAGTTTGACAACCG |
|  | MPN310-dup5-R | **TTGGCGCTTAAAGCC**GGCTA |
| Mutagenesis sites for the internal start codon of P28 were underlined (ATG → TTA). | | |
| Overlapped regions for cloning were indicated by bold characters  *Not*I site was enclosed in a box | | |
| The same primers were used for the construction of pKM310-inc_5 and pKM310-inc_5+5 | | |
