# Supplementary material for: Molecular ruler of the attachment organelle in Mycoplasma pneumoniae
Source: PLoS Pathog. 2021 Jun 10;17(6):e1009621. doi: 10.1371/journal.ppat.1009621 (PMC8191905; doi:10.1371/journal.ppat.1009621)
Supplement: S2 Table — (DOCX) [file ppat.1009621.s002.docx]

| **S2 Table**  Plasmids used in this study | | | | | |
| --- | --- | --- | --- | --- | --- |
| Plasmid | Marker | Replicon (Vector) | Promotor | Gene | Reference |
| pKV104 | Ap^r^ Cm^r^ |  |  | Tn*4001*Cm | [42] |
| pMPN310-E | Km^r^ | pENTR/D-TOPO |  | *hmw2* | [40] |
| pmCherry | Ap^r^ | pUC19 |  | *mCherry* |  |
| pKM310-mCh | Km^r^ | pMPN310-E |  | *mCherry-hmw2* | This study |
| pKM310-standard | Km^r^ | pKM310-E |  | *mCherry-hmw2(*Δ*p28)* | This study |
| pKM310-dec_4 | Km^r^ | pKM310-standard |  | *mCherry-d4* | This study |
| pKM310-dec_5 | Km^r^ | pKM310-standard |  | *mCherry-d5* | This study |
| pKM310-dec_6 | Km^r^ | pKM310-standard |  | *mCherry-d6* | This study |
| pKM310-dec_78 | Km^r^ | pKM310-standard |  | *mCherry-d78* | This study |
| pKM310-dec_9 | Km^r^ | pKM310-standard |  | *mCherry-d9* | This study |
| pKM310-dec_9α | Km^r^ | pKM310-standard |  | *mCherry-d9a* | This study |
| pKM310-inc_4 | Km^r^ | pKM310-standard |  | *mCherry-dup4* | This study |
| pKM310-inc_5 | Km^r^ | pKM310-standard |  | *mCherry-dup5* | This study |
| pKM310-inc_5+5 | Km^r^ | pKM310-standard |  | *mCherry-dup55* | This study |
| pTK170-D | Ap^r^ Gm^r^ | pISM2062.2 | *tuf* | Tn*4001*Gm(*eyfp*) | [9] |
| pKM170-standard | Ap^r^ Gm^r^ | pTK-170-D | *tuf* | Tn*4001*Gm(*mCherry-hmw2(*Δ*p28)-eyfp*) | This study |
| pKM170-dec_4 | Ap^r^ Gm^r^ | pTK-170-D | *tuf* | Tn*4001*Gm(*mCherry-d4-eyfp*) | This study |
| pKM170-dec_5 | Ap^r^ Gm^r^ | pTK-170-D | *tuf* | Tn*4001*Gm(*mCherry-d5-eyfp*) | This study |
| pKM170-dec_6 | Ap^r^ Gm^r^ | pTK-170-D | *tuf* | Tn*4001*Gm(*mCherry-d6-eyfp*) | This study |
| pKM170-dec_78 | Ap^r^ Gm^r^ | pTK-170-D | *tuf* | Tn*4001*Gm(*mCherry-d78-eyfp*) | This study |
| pKM170-dec_9 | Ap^r^ Gm^r^ | pTK-170-D | *tuf* | Tn*4001*Gm(*mCherry-d9-eyfp*) | This study |
| pKM170-dec_9α | Ap^r^ Gm^r^ | pTK-170-D | *tuf* | Tn*4001*Gm(*mCherry-d9a-eyfp*) | This study |
| pKM170-inc_4 | Ap^r^ Gm^r^ | pTK-170-D | *tuf* | Tn*4001*Gm(*mCherry-dup4-eyfp*) | This study |
| pKM170-inc_5 | Ap^r^ Gm^r^ | pTK-170-D | *tuf* | Tn*4001*Gm(*mCherry-dup5-eyfp*) | This study |
| pKM170-inc_5+5 | Ap^r^ Gm^r^ | pTK-170-D | *tuf* | Tn*4001*Gm(*mCherry-dup55-eyfp*) | This study |
