# Supplementary material for: Molecular ruler of the attachment organelle in Mycoplasma pneumoniae
Source: PLoS Pathog. 2021 Jun 10;17(6):e1009621. doi: 10.1371/journal.ppat.1009621 (PMC8191905; doi:10.1371/journal.ppat.1009621)
Supplement: S3 Table — (DOCX) [file ppat.1009621.s003.docx]

| **S3 Table**  Size-modified HMW2-derivative mutants in this study and their characterization | | | | | | | | | |
| --- | --- | --- | --- | --- | --- | --- | --- | --- | --- |
| Strain | Genotype | Full length of  HMW2 derivatives* | | Coiled-coil domain | | | EYFP and mCherry | Cytoskeletal core (EM) | Gliding motility |
|  |  | Length (aa) | Change (aa) | Length (aa) | Change (aa) | Esti- mation (nm) | Distance (nm) | Bold type (nm) | Speed (nm/s) |
| M129 | Wild type | 1818 | 0 | 1249 | 0 | 187 | nd | 187±8 | 195±40 |
| NA7 | M129 *hmw2*::Tn*4001*Cm | - | - | - | - | - | - | - | - |
| Standard | NA7 (pKM170-standard) | 1818 | 0 | 1249 | 0 | 187 | 185±31 | 183±7 | 182±36 |
| dec_4 | NA7 (pKM170-dec_4) | 1521 | -297 | 981 | -268 | 147 | 153±25 | 148±15 | 150±39 |
| dec_5 | NA7 (pKM170-dec_5) | 1527 | -291 | 992 | -257 | 149 | 152±32 | 151±18 | 156±38 |
| dec_6 | NA7 (pKM170-dec_6) | 1580 | -238 | 1069 | -180 | 160 | 164±31 | 165±21 | 151±32 |
| dec_78 | NA7 (pKM170-dec_78) | 1502 | -316 | 1088 | -161 | 163 | 161±32 | 151±12 | 168±40 |
| dec_9 | NA7 (pKM170-dec_9) | 1661 | -157 | 1145 | -104 | 172 | 170±29 | 167±15 | 186±33 |
| dec_9α | NA7 (pKM170-dec_9α) | 1593 | -225 | 1145 | -104 | 172 | 171±33 | 163±16 | 186±43 |
| inc_4 | NA7 (pKM170-inc_4) | 2083 | +265 | 1517 | +268 | 228 | 97±41 | 218±17 | 208±32 |
| inc_5 | NA7 (pKM170-inc_5) | 2096 | +278 | 1506 | +257 | 226 | 222±35 | 225±18 | 215±27 |
| inc_5+5 | NA7 (pKM170-inc_5+5) | 2373 | +555 | 1763 | +514 | 265 | 257±46 | 270±25 | 211±26 |

The length of HMW2 derivatives did not include the amino acid numbers of mCherry and EYFP.
